# Identification of a novel autophagy signature for predicting survival in patients with lung adenocarcinoma

**DOI:** 10.7717/peerj.11074

**Published:** 2021-04-21

**Authors:** Jin Duan, Youming Lei, Guoli Lv, Yinqiang Liu, Wei Zhao, Qingmei Yang, Xiaona Su, Zhijian Song, Leilei Lu, Yunfei Shi

**Affiliations:** 1 Department of Geriatric Thoracic Surgery, The First Hospital of Kunming Medical University, Kunming City, Yunnan Province, P.R. China; 2 Department of Cancer Center, Daping Hospital, Army Medical University, Chongqing, China; 3 Origimed Co. Ltd., Shanghai, P.R. China

**Keywords:** Lung adenocarcinoma, LASSO Cox regression, The Cancer Genome Atlas, Gene set enrichment analysis, Immune cell analysis, Autophagy, Gene expression omnibus database, Multivariate cox regression analyses, Prognosis, Molecular biomarkers

## Abstract

**Background:**

Lung adenocarcinoma (LUAD) is the most commonhistological lung cancer subtype, with an overall five-year survivalrate of only 17%. In this study, we aimed to identify autophagy-related genes (ARGs) and develop an LUAD prognostic signature.

**Methods:**

In this study, we obtained ARGs from three databases and downloaded gene expression profiles from The Cancer Genome Atlas (TCGA) and Gene Expression Omnibus (GEO) database. We used TCGA-LUAD (*n* = 490) for a training and testing dataset, and GSE50081 (*n* = 127) as the external validation dataset.The least absolute shrinkage and selection operator (LASSO) Cox and multivariate Cox regression models were used to generate an autophagy-related signature. We performed gene set enrichment analysis (GSEA) and immune cell analysis between the high- and low-risk groups. A nomogram was built to guide the individual treatment for LUAD patients.

**Results:**

We identified a total of 83 differentially expressed ARGs (DEARGs) from the TCGA-LUAD dataset, including 33 upregulated DEARGs and 50 downregulated DEARGs, both with thresholds of adjusted *P* < 0.05 and |Fold change| > 1.5. Using LASSO and multivariate Cox regression analyses, we identified 10 ARGs that we used to build a prognostic signature with areas under the curve (AUCs) of 0.705, 0.715, and 0.778 at 1, 3, and 5 years, respectively. Using the risk score formula, the LUAD patients were divided into low- or high-risk groups. Our GSEA results suggested that the low-risk group were enriched in metabolism and immune-related pathways, while the high-risk group was involved in tumorigenesis and tumor progression pathways. Immune cell analysis revealed that, when compared to the high-risk group, the low-risk group had a lower cell fraction of M0- and M1- macrophages, and higher CD4 and PD-L1 expression levels.

**Conclusion:**

Our identified robust signature may provide novel insight into underlying autophagy mechanisms as well as therapeutic strategies for LUAD treatment.

## Introduction

Lung cancer is the leading cause of deaths from malignant tumors worldwide, with an estimated 228,820 new cases and 135,720 deaths in the United States in 2020 (Siegel, Miller & Jemal, 2020). Non-small-cell lung cancer (NSCLC), one of the major histological subtypes, accounts for approximately 80–85% of all lung cancer cases (Molina et al., 2008). NSCLC can be further classified into three types: squamous-cell carcinoma, adenocarcinoma, and large-cell carcinoma (Petersen, 2011). Lung adenocarcinoma (LUAD) is the most common histological subtype, accounting for more than 40% of all lung cancer cases (Shi et al., 2016). Despite substantial efforts devoted to LUAD diagnosis and treatment, the overall five-year survival rate for this disease is still relatively low at 17% (Miller et al., 2019). This poor prognosis is largely due to the lack of reliable biomarkers that could predict patient survival in the early stages. Therefore, there is emerging interest in identifying novel molecular biomarkers that could improve the prognosis and therapeutic strategies for LUAD patients.

Autophagy, a protective self-cannibalization process, is thought to facilitate the degradation and recycling of cytoplasmic material in order to maintain cellular homeostasis. In recent years, growing evidence has supported that autophagy is linked to a variety of cancers and pathological infectious and neurodegenerative diseases (Levine, Packer & Codogno, 2015). However, autophagy’s definitive role in tumorigenesis onset and progression remains inconclusive. It is currently recognized that autophagy plays a dual role in cancer by inhibiting tumor development in the early stage and promoting tumor progression, and even making tumor cells drug-resistant, in the advanced stage. Previous studies have investigated the role of several autophagy-related genes (ARGs) in the development and progression of lung cancer (Jaboin, Hwang & Lu, 2009). It was found that high Nrf2 expression can promote NSCLC progression by activating autophagy, which is also known to facilitate resistance to cisplatin-based therapy by activating the AMPK/mTOR signaling pathway in lung adenocarcinoma (Wu et al., 2015). These studies attempted to explore the role of ARGs in LUAD progression, but little effort has been made to investigate their role in lung adenocarcinoma prognosis using global expression patterns. Exploring the appropriate molecular autophagy biomarkers may be important in the fight against LUAD.

In this study, we downloaded LUAD datasets from The Cancer Genome Atlas (TCGA) and the Gene Expression Omnibus (GEO) database to establish a comprehensive signature based on ARGs in order to predict survival outcome in LUAD patients. We screened differentially expressed autophagy-related genes (DEARG) from the TCGA-LUAD dataset. Subsequently, we performed GO and KEGG enrichment analyses to show the top enriched terms across the DEARGs. Using the least absolute shrinkage and selection operator (LASSO) and multivariate Cox regression analyses, we developed a robust autophagy signature related to survival outcomes in both TCGA and validated GEO datasets. We conducted gene set enrichment analysis (GSEA) and immune cell analysis to compare the perturbed pathways and immune phenotypes between the low risk-group and high risk-group. Finally, a prognostic nomogram was established by incorporating the risk score and clinicopathologic factors. In summary, the autophagy signature from our study may serve as a promising biomarker signature for monitoring the prognosis of LUAD patients.

## Material and Methods

### The flowchart and data acquisition

**Figure 1:**
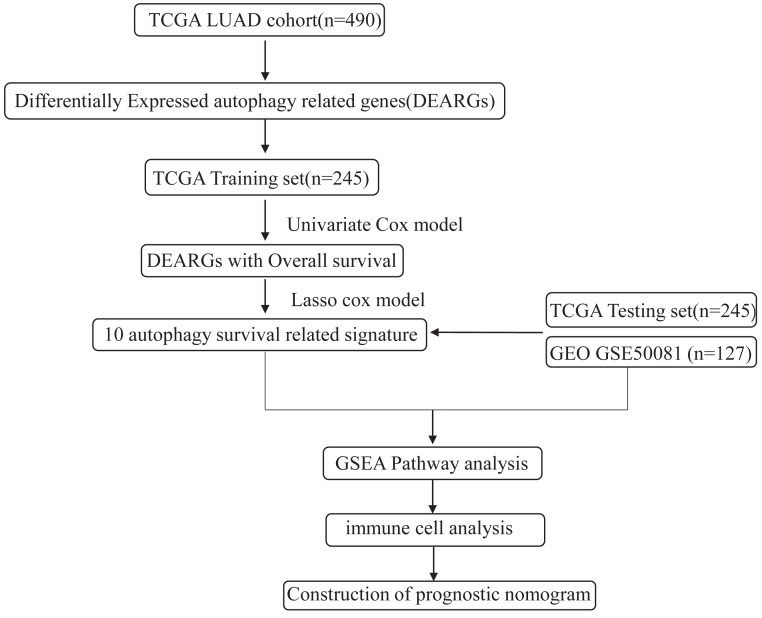
An overview of identification of LUAD prognostic related autophagy signature in our study.

The workflow of this study is shown in Fig. 1. We downloaded LUAD mRNA sequencing data (level 3) and their corresponding clinical patient data from TGGA (https://cancergenome.nih.gov/). Only the samples with complete clinicopathological information and more than 30 days of overall survival (OS) were included in this study. We used GSE50081 from the GEO database as the external validation dataset. Overall, 490 patients were randomly assigned into a training cohort (*n* = 245) and a testing cohort (*n* = 245) to satisfy the following criteria: (1) samples were randomly divided into training and testing datasets; and (2) gender, age, and clinical stage distributions between the two groups looked similar (Table 1). In addition, another 127 samples from GSE50081 were used as the validation dataset. This study was conducted in accordance with TCGA publication guidelines (http://cancergenome.nih.gov/publications/publicationguidelines).

**Table 1:** Summary of TCGA LUAD patient demographics and characteristics.

Character	Training cohort (*n* = 245)	Testing cohort (*n* = 245)
	No. of patients %	No. of patients %
Age (mean, range)	65.0(33–88)	65.4(38-87)
Gender		
Male	118(48.2)	135(55.1)
Female	126(51.4)	110(44.9)
Stage		
I	122(49.8)	141(57.6)
II	63(25.7)	52(21.2)
III	43(17.6)	36(14.7)
IV	14(5.7)	11(4.5)

### ARG set curation

ARGs were curated from the Human Autophagy Database (HADb, http://www.autophagy.lu/index.html), REACTOME AUTOPHAGY in the Molecular Signatures Database v6.2 (MSigDB, http://software.broadinstitute.org/gsea/msigdb), and genes with “autophagy” term relevance scores > =7 from the GeneCards website (https://www.genecards.org/). After eliminating the overlapping genes, these three gene sets were combined and integrated into an autophagy-associated gene set. The ARG list was comprised of 366 genes when finally constructed.

### ARG differential expression analysis

We downloaded all the genes of the LUAD samples from TCGA database in FPKM format, which we then converted to TPM format using the formula: TPM = 10^6^ ×FPKM/sum(FPKM). All expression profiles were converted to [log2(TPM + 1)]. We used the “Limma” package (Ritchie et al., 2015) in R software to identify the differentially expressed genes (DEGs) between tumor and normal tissue. The Benjamini–Hochberg method was used to adjust *p* values, and we considered adjusted *P* < 0.05 and fold change (FC) > 1.5 as the cutoff criterion for DEG identification. The intersection of DEGs and ARGs was considered the set of significant DEARGs for further analysis. Additionally, we performed volcano plot and heatmap analysis to screen the common DEARGs across the datasets.

### Kyoto Encyclopedia of Genes and Genomes and gene ontology analysis

We analyzed the function of significant DEARGs using the Kyoto Encyclopedia of Genes and Genomes (KEGG), gene ontology (GO) functional enrichment analyses (Ashburner et al., 2000), and the Database for Annotation, Visualization, and Integrated Discovery (DAVID, http://david.ncifcrf.gov/; Jiao et al., 2012). A *P* value of <0.05 was considered statistically significant.

### Prognostic model construction and performance assessment

We first conducted univariate Cox proportional hazard regression to identify the DEARGs that were significantly associated with overall survival (*P* value < 0.05) in the training cohort using the survival package (http://bioconductor.org/packages/survival/) in R. The LASSO Cox regression method (Sauerbrei, Royston & Binder, 2007) was then employed to select optimal gene combination variables, and then the “glmnet” package in R was used to construct the risk signature. Only genes with non-zero coefficients in the LASSO model were put into the multivariate Cox regression model to calculate the risk score. The prognostic model formula was as follows: Risk score = (expr gene_1_ ×*β*_1_) + (expr gene_2_ ×*β*_2_) + ⋯ + (expr gene_*n*_ × *β*_*n*_), where “expr” represents the gene_*i*_ expression value and “ *β*_*i*_” represents the estimated regression gene_*i*_ coefficient.

Using the median risk score value as a cutoff, we divided the LUAD patients into low-risk and high-risk groups. We employed the Kaplan–Meier (K-M) survival curves to show the OS differences between the high-risk and low-risk groups. We used the area under the curve (AUC) of the time-dependent receiver operating characteristic (ROC) curve and the R package survivalROC to evaluate the risk signature’s efficiency. These analyses were conducted in the TCGA testing dataset and GEO datasets.

### GSEA

GSEA (version 3.0, http://www.broadinstitute.org/gsea/index.jsp) was used to evaluate the biological pathways or gene sets that differed significantly between the high-risk and low-risk groups. The parameters were as follows: max gene set size of 500, min size of 15, number of permutations of 1,000, and enriched gene sets with a nominal *P* value < 0.05 were considered significant.

### Immune cell analysis

CIBERSORT (Newman et al., 2015), a deconvolution algorithm, was employed to estimate the relative abundance of immune-infiltrating cell composition in tissues based on their expression profiles. We submitted the LUAD gene expression dataset to the CIBERSORT website (http://cibersort.stanford.edu/) and used LM22 (22 immune cell types) as the signature gene file. The program was implemented with 1,000 permutations. Next, the output values generated by CIBERSORT were defined as immune cell infiltration fractions per sample. The output results were used to compare immune cell infiltration fractions across low-risk and high-risk patients.

### Nomogram construction and validation

A prognostic nomogram was constructed to combine ARG signatures and other clinicopathological factors using the “rms” package (https://cran.r-project.org/web/packages/rms/index.html) in R. To evaluate the accuracy of the nomogram, we applied calibration curves and K-M analysis to compare the concordance between the predicted survival and observed survival.

### Statistical analysis

All statistical analyses in our study were conducted using R language (version 3.5.1, https://www.r-project.org/). Boxplots and violin plots were generated using the “ggplot2” package in R language. *P* < 0.05 was considered significant.

## Results

### Identification of autophagy-related risk signature in the LUAD training cohort

**Figure 2:**
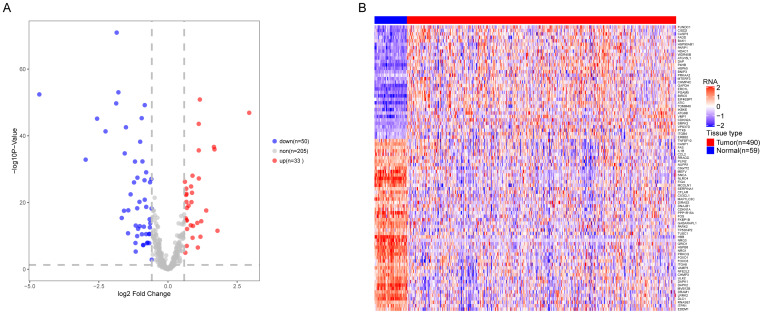
Differential expression of autophagy-related genes(ARGs) in TCGA-LUAD. The differential expression of ARGs in LUAD (*n* = 490) compared with normal Lung tissues (*n* = 59) was shown in the volcano plot and heatmap plot. (A) In the volcano plot, red dots represent upregulated DEARGs, and blue dots represent downregulated DEARGs, and the gray dots represent the ARGs which are not differentially expressed. (B) The heatmap plot demonstrates differentially expressed genes between LUAD and normal Lung tissues. Red color is high expressed and blue color is low expressed.

**Figure 3:**
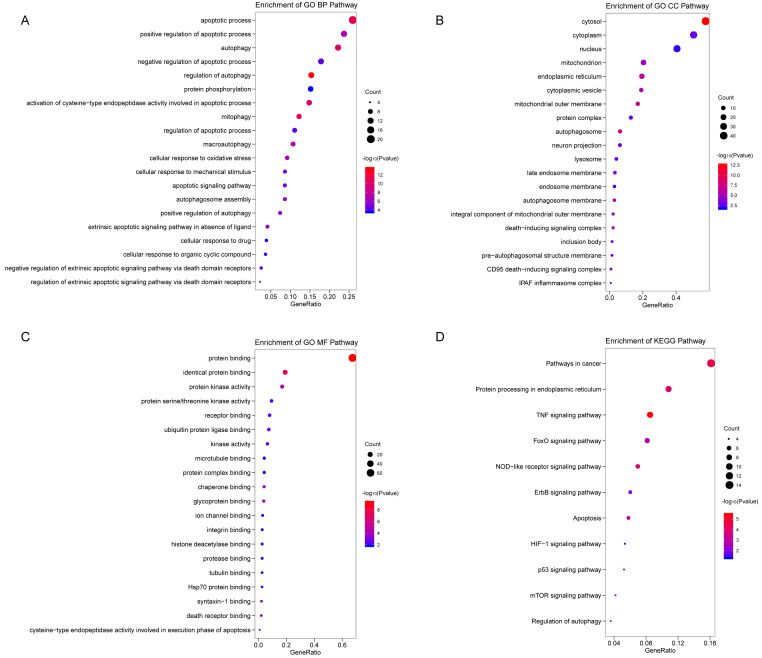
Functional KEGG and GO analysis for Differentially expressed autophagy genes. The vertical axis represents GO or KEGG pathway annotations. The horizontal axis GeneRatio represents the ratio of the numbers of differential autophagy genes enriched in the pathway to the total number of genes in the pathway (A) The top 20 significant terms of CC. (B) The top 20 significant terms of BP. (C) The top 20 significant terms of MF. (D) The top 11 significant terms of KEGG pathways. BP, biological process; CC, cellular component; MF, molecular function.

We analyzed the expression of 366 ARGs in 490 LUAD and 59 normal lung tissue samples using the “limma” package in R software. In total, we identified 83 DEARGs from the LUAD samples, including 33 upregulated DEARGs and 50 downregulated DEARGs with a cutoff criteria of adjusted *P* < 0.05, |FC| > 1.5. The volcano plot and heatmap for these 83 DEARGs in normal and tumor tissues are displayed in Fig. 2. Additionally, to better explore the biological interpretation of these DEARGs, we performed functional enrichment pathway analyses. According to the GO enrichment analysis results, we found that these DEARGs were primarily involved in autophagy, protein binding, and autophagosome related to biological process (BP), molecular function (MF), and cellular component (CC) terms (Figs. 3A–3C). Moreover, KEGG enrichment analysis also indicated that these genes primarily participated in cancer pathways, protein processing in the endoplasmic reticulum, as well as the TNF signaling pathway (Fig. 3D).

### Identifying prognostic risk DEARGs in the LUAD training set

**Figure 4:**
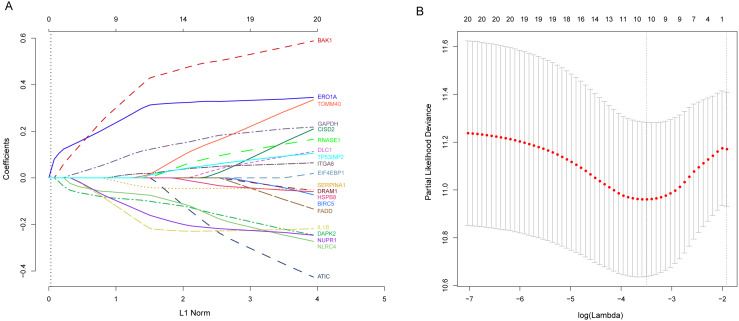
Identification of autophagy-related signature associated overall survival (OS) by LASSO cox regression. (A) LASSO coefficient of 10 prognostic DEARGs by 10-fold cross-validation. (B) Partial likelihood deviance with corresponding log (*λ*) values at the minimal deviance of the model.

Next, we performed univariate Cox proportional hazards regression analysis to identify prognostic DEARGs in the LUAD training set using the coxph function of the survival package in R (Zhang, 2002). We found a total of 20 DEARGs that were significantly associated with OS (*P* <  = 0.05, Table 2). Moreover, LASSO Cox regression was subsequently used to avoid overfitting problems in the risk signature. Ten key autophagy-related genes (BAK1, DAPK2, ERO1A, GAPDH, IL1B, ITGA6, NLRC4, NUPR1, SERPINA1, and TOMM40) were retained when the optimal lambda value was achieved (Figs. 4A–4B). Finally, an autophagy-related signature was established using multivariate Cox regression and the following risk score formula for each patient was as follows:

\begin{eqnarray*}\text{Risk score}=0.49915\times (\text{expression value of BAK1})+(-0.10340)\nonumber\\\displaystyle \quad \times (\text{expression value of DAPK2})+0.36101\times (\text{expression value of ERO1A})\nonumber\\\displaystyle \quad +0.15103\times (\text{expression value of GAPDH})+(-0.26998)\nonumber\\\displaystyle \quad \times (\text{expression value of IL1B})+0.02068\times (\text{expression value of ITGA6})\nonumber\\\displaystyle \quad +(-0.08339)\times (\text{expression value of NLRC4})+(-0.19307)\nonumber\\\displaystyle \quad \times (\text{expression value of NUPR1})+(-0.05327)\times (\text{expression value of SERPINA1})\nonumber\\\displaystyle \quad +0.02443\times (\text{expression value of TOMM40}). \end{eqnarray*}

**Table 2:** Top 20 DEARGs significantly associated with the OS of patients with LUAD (*P* < 0.05).

Gene	HR	HR lower 95% CI	HR lower 95% CI	*P*-value
ERO1A	1.56196	1.25909	1.93768	0.00006
DAPK2	0.60780	0.45809	0.80643	0.00030
GAPDH	1.59982	1.24115	2.06213	0.00031
BAK1	1.78522	1.26329	2.52278	0.00087
BIRC5	1.28311	1.08715	1.51439	0.00253
RNASE1	0.81448	0.70417	0.94206	0.00607
ITGA6	1.23424	1.05888	1.43864	0.00948
NLRC4	0.66828	0.48535	0.92017	0.01102
TOMM40	1.41904	1.09157	1.84476	0.01241
DLC1	0.81861	0.69603	0.96277	0.01490
ATIC	1.57720	1.07702	2.30968	0.01705
EIF4EBP1	1.26109	1.04204	1.52619	0.01857
FADD	1.58377	1.06979	2.34469	0.01960
CISD2	1.64453	1.06527	2.53878	0.02558
TP53INP2	1.29654	1.02516	1.63975	0.02876
NUPR1	0.82506	0.69025	0.98620	0.03631
SERPINA1	0.88466	0.78856	0.99248	0.03919
HSPB8	0.82499	0.68862	0.98836	0.03945
IL1B	0.82574	0.68334	0.99781	0.04346
DRAM1	0.83223	0.69074	1.00270	0.05087

**Figure 5:**
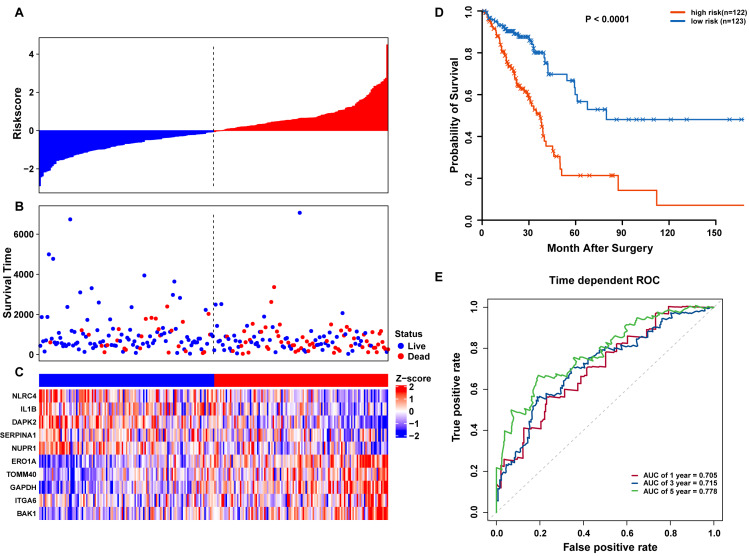
OS-related prognostic model of LUAD patients in TCGA training set. (A–C) The prognostic model distribution of risk score, survival status, and the heatmap of autophagy-related signature. (A) Risk score distribution curves; (B) the survival time and status of LUAD patient; (C) a heatmap which displays the normalized *z*-score of TPM values for 10 genes in autophagy-related signature. Red indicates higher expression and blue indicates lower expression. (D) Kaplan-Meier curves of the prognostic predictors for high-risk and low-risk patients with LUAD. (E) Time-dependent ROC curves for evaluating the accuracy of the risk scores.

Risk scores for each patient were calculated, and the patients in the training set were divided into high-risk (*n* = 122) and low-risk groups (*n* = 123), according to the median risk score cutoff. In the training set, we determined the risk score distribution, OS status, and the corresponding expression profiles of 10 ARGs (Figs. 5A–5C). The heatmap showed that patients in the high-risk group tended to have higher expression patterns of risky ARGs (ERO1A, TOMM40, GAPDH, ITGA6, and BAK1). On the other hand, patients in the low-risk group tended to have higher expression patterns of protective autophagy genes (NLRC4, IL1B, DAPK2, SERPINA1, and NUPR1) (Fig. 5C). Moreover, the K-M survival curve and the log-rank test exhibited that patients in the high-risk group had a significantly shorter OS time than those in the low-risk group (median time = 2.15 years vs. 2.91 years, respectively, *p* < 0.001; Fig. 5D). Additionally, we evaluated the predictive performance of the risk signature model with these prognostic biomarkers using time-dependent ROC curves. The area under the ROC curves for one, three, and five-year OS predictions of the risk scores were 0.705, 0.715, and 0.778 (Fig. 5E), respectively.

### Validation of the autophagy signature in TCGA and GEO datasets

**Figure 6:**
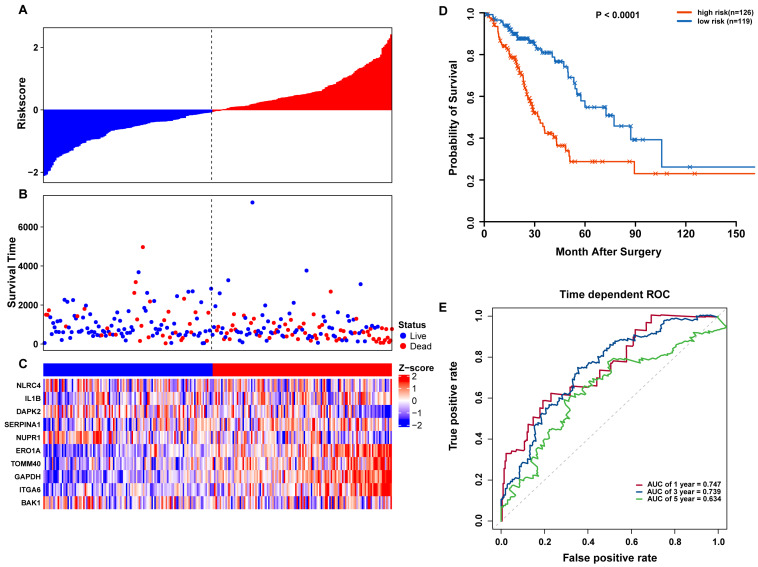
OS-related prognostic model of LUAD patients in TCGA testing set. (A–C) The prognostic model distribution of risk score, survival status, and the heatmap of autophagy-related signature. (A) Risk score distribution curves; (B) the survival time and status of LUAD patients; (C) a heatmap which displays the normalized *z*-score of TPM values for 10 genes in autophagy-related signature. Red indicates higher expression and blue indicates lower expression. (D) Kaplan-Meier curves of the prognostic predictors for high-risk and low-risk patients with LUAD. (E) Time-dependent ROC curves for evaluating the accuracy of the risk scores.

To confirm our findings, we performed additional testing and used external validation datasets to assess the predictive performance of the 10-gene autophagy signature. First, we validated our autophagy-related signature using a TCGA testing set as an internal validation series. A total of 245 LUAD samples were collected and used to assess the risk signature’s performance. Using the same risk score cutoff, we classified the patients into high-risk (*n* = 126) and low-risk (*n* = 119) groups in the internal testing set. In accordance with our previous findings, the distribution of risk score, OS status, and ARG expression were similar as those in the training set (Figs. 6A–6C). Moreover, patients with higher risk scores had significantly shorter median OS than those with lower risk scores (log-rank test *P* < 0.001; Fig. 6D). The AUCs for one, three, and five-year OS predictions for the risk scores were 0.747, 0.739, and 0.634, respectively, which results were similar to the training set (Fig. 6E).

**Figure 7:**
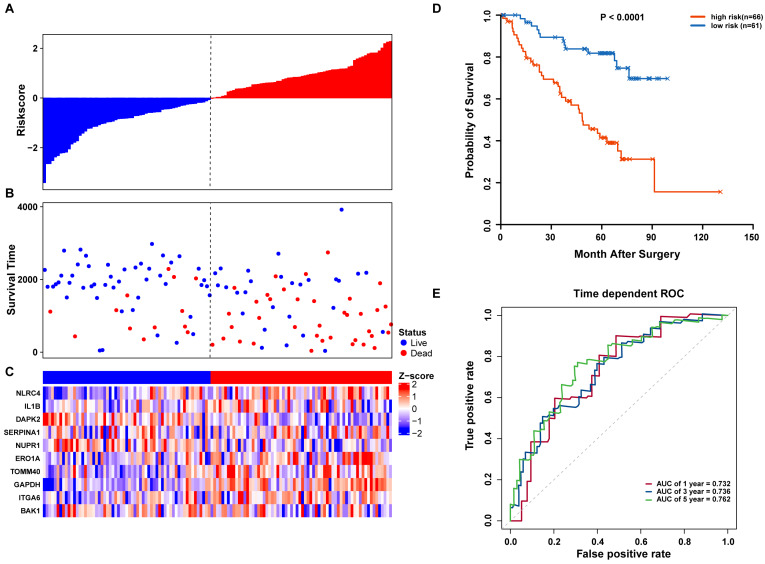
OS-related prognostic model of LUAD patients in GSE50081 set. (A–C) The prognostic model distribution of risk score, survival status, and the heatmap of autophagy-related signature. (A) Risk score distribution curves; (B) the survival time and status of LUAD patients; (C) a heatmap which displays the normalized *z*-score of TPM values for 10 genes in autophagy-related signature. Red indicates higher expression and blue indicates lower expression. (D) Kaplan-Meier curves of the prognostic predictors for high-risk and low-risk patients with LUAD. (E) Time-dependent ROC curves for evaluating the accuracy of the risk scores.

We further validated our autophagy signature using another independent data set obtained from GSE50081. The distribution of risk score, OS status, and ARG expression in the testing dataset are shown in Figs. 7A–7C. The results confirmed our model’s ability to predict survival. The 10-autophagy-related signature model could effectively predict the OS in patients from the GSE50081 dataset (log-rank test *P* =  < 0.0001; Fig. 7D). The AUC values for the one, three, and five-year OS models were 0.732, 0.736, and 0.762, respectively (Fig. 7E). These results confirmed that the autophagy-related signature could accurately predict the OS of LUAD patients.

### GSEA of high-risk and low-risk LUAD patient characteristics

**Figure 8:**
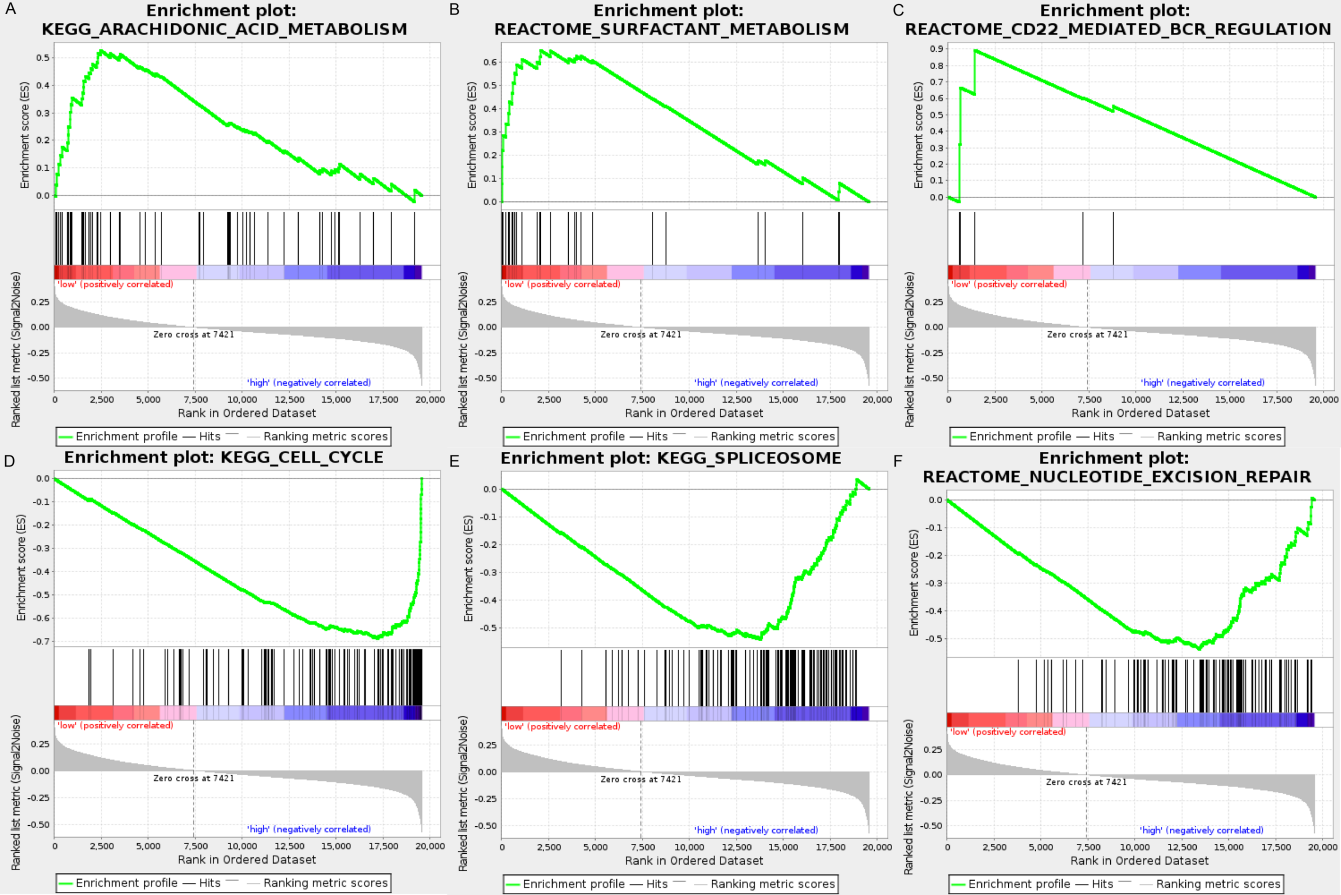
The GSEA analysis results in TCGA LUAD. (A) “Arachidonic acid metabolism”, (B) “Surfactant metabolism”, (C) “CD22 mediated BCR regulation”, (D) “Cell cycle”, (E) “Spliceosome”, (F) “Nucleotide excision repair”.

We carried out GSEA to explore the high-risk and low-risk groups’ biological processes and signaling pathways associated with the autophagy signature. We compared the gene expression profiles of high-risk and low-risk LUAD patients that were classified by the 10-autophagy-related gene signature in both the training set and testing set. The GSEA results revealed that the genes in the low-risk group were closely associated with several metabolism and immune-related pathways, including arachidonic acid metabolism (NES = 1.65, *P* = 0.014), surfactant metabolism (NES = 1.63, *P* = 0.028), and the CD22-mediated BCR regulation pathway (NES = 1.58, *P* = 0.010). Genes in the high risk-group were enriched in several tumor progression pathways, including cell cycle (NES = −2.16, *P* = 0), spliceosomes (NES = −2.10, *P* = 0), and nucleotide excision repair (NES =- 2.09, *P* = 0). The GSEA results are shown in Fig. 8.

### Distinct immune phenotype characterization of high-risk and low-risk LUAD patients

**Figure 9:**
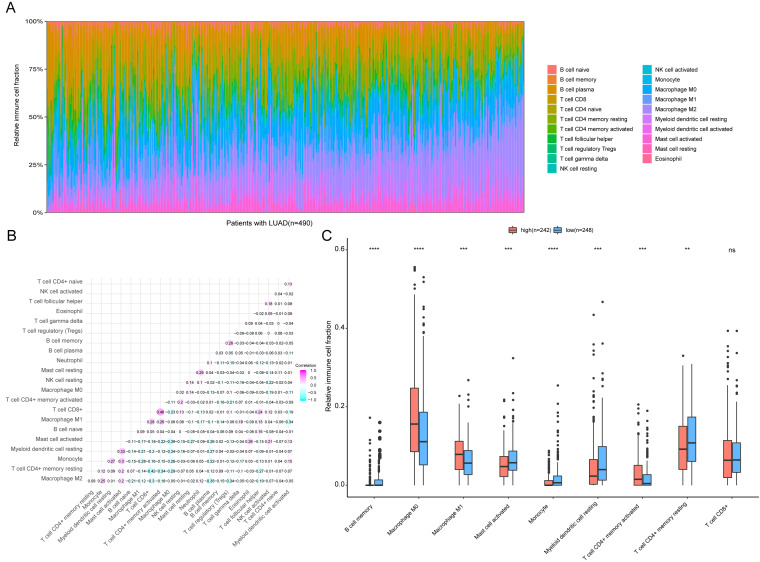
Immune profiling between high-risk and low-risk patients with LUAD. (A) The relative proportions of 22 immune infiltrating cell in patients with TCGA LUAD. (B) Correlation matrix for relative proportions of the 22 immune cell types. (C) Box plots of immune cell infiltration proportion between high- and low-risk groups. * *P* < 0.05, ** *P* < 0.01, *** *P* < 0.001, **** *P* < 0.0001.

**Figure 10:**
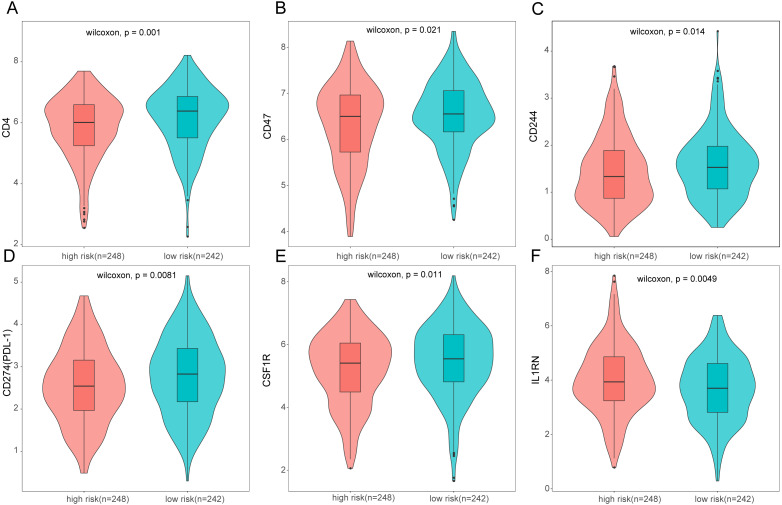
Expression of immune associated genes between low-risk and high-risk groups. Statistic differences between groups were calculated by Wilcoxon test.

To further analyze the association between the ARGs and the tumor immune microenvironment, we used CIBERSORT software to estimate the infiltration fraction across the 22 distinct immune cell types in LUAD patients. The distribution of the 22 immune cell types in each individual are shown in Fig. 9A. The relative proportions of the 22 immune cell types were found to be weakly to moderately correlated (Fig. 9B). Additionally, we intensively investigated the potential differences between the low-risk and high-risk groups. In the high-risk group, we observed that the relative fraction of M0- and M1- macrophages and T cell CD4+ memory activated were significantly increased, while the relative fraction of Myeloid dendritic cells, Mast cells activated, and T cell CD4+ memory resting were significantly decreased (Fig. 9C). However, we also found no significant differences in CD8 T cell infiltration between these two groups (Fig. 9C). To investigate the immune status of the LUAD tumors, we selected immune checkpoints (PD-L1) and other immune-related genes (including CD4, CD47, CD244, CSF1R, and IL1RN) to explore the differences between the high-risk and low-risk groups. Compared to the high-risk group, we found that CD4, CD244, PD-L1, CSF1R, and CD47 were significantly overexpressed in low-risk patients (*P* < 0.05, Figs. 10A–10F). Moreover, classic immune checkpoints such as PDCD1(PD-1), CTLA-4, HAVCR2(Tim-3), LAG3, and TIGIT were also compared between the high-risk and low-risk groups, but no significant differences were found (Fig. S1). Taken together, these data implied that the autophagy signature may serve as an indicator of LUAD immune status.

### Nomogram construction and validation

**Figure 11:**
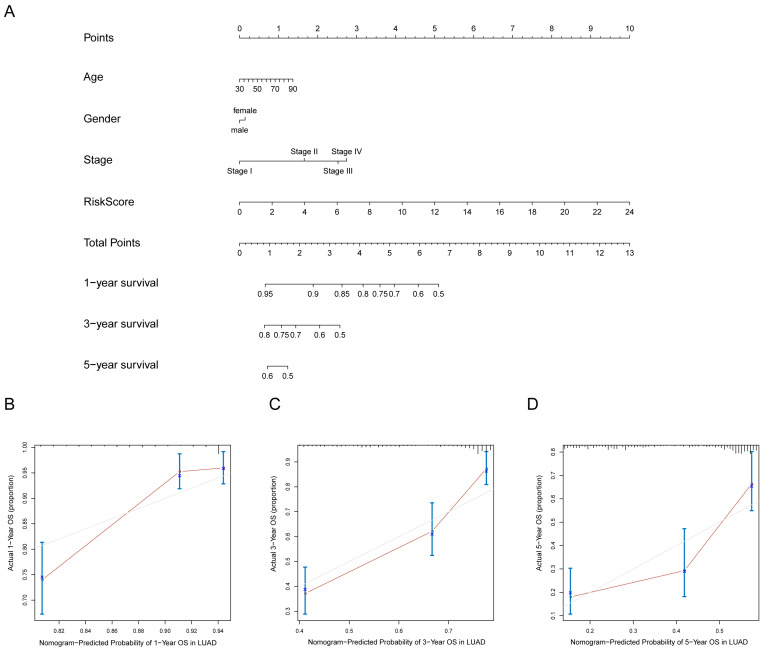
Nomogram for predicting the 1-,3-, 5-year survival with risk score. (A) Prognostic nomogram for LUAD patients in TCGA; (B-D) Calibration curves for the nomogram at 1-,3-,5-year.

A prognostic nomogram can quantitatively predict an individual’s risk by integrating autophagy signature risk scores and clinicopathologic features. The training and testing dataset, both from TCGA, were combined to construct nomograms for validation. We constructed a nomogram to predict OS by incorporating the risk scores with age, gender, and tumor, node, metastasis (TNM) stage. Each variable was assigned points in proportion to its risk contribution to survival, and the C-index to evaluate the OS of the model was 0.721(Fig. 11A). The calibration curves suggested agreement between the actual and predicted OS (Figs. 11B–11D).

## Discussion

LUAD remains the most common and aggressive type of lung cancer worldwide. The TNM classification system is routinely used for cancer staging and LUAD prognosis (Woodard, Jones & Jablons, 2016). However, heterogeneous prognostic outcomes and different treatment responses still exist for patients at the same stage. Therefore, reliable and accurate molecular biomarkers or models for LUAD prognosis are urgently required. Autophagy is a protective process that plays a crucial role in responding to cellular stress and maintaining cellular homeostasis. It is involved in and regulated by a series of genes that are closely related to various cellular degradation processes and biochemical reactions. In recent years, cumulative evidence has indicated that autophagy acts as a “double-edged sword” by suppressing tumors at the initial stage while causing tumor progression, and consequently drug resistance, in the later stages. Several studies about autophagy-related prognostic signatures in colorectal cancer (Zhou et al., 2019), NSCLC (Liu et al., 2019; Zhu, Wang & Hu, 2020), serous ovarian cancer (An et al., 2018), and prostate cancer (Hu et al., 2020) have provided abundant support of the link between autophagy and tumorigenesis. However, there have been no systematic analyses of autophagy-based signatures for LUAD. This is the first systematic analysis of ARGs associated with the OS of LUAD patients using large clinical datasets.

In this study, we used two datasets (TCGA and GEO) to explore the associations between ARGs and LUAD prognosis.

First, we screened differentially expressed autophagy-related genes from TCGA LUAD dataset and identified 83 DEARGs, 33 of which were upregulated and 50 that were downregulated. GO and KEGG enrichment analyses were conducted to confirm that the top enriched terms were involved in the cancer autophagy process. In addition, KEGG analysis showed enrichment in the cancer pathways, protein processing in the endoplasmic reticulum, and the TNF signaling pathway, which suggested that autophagy gene dysregulation may participate in cancer biological processes. Using LASSO and multivariate Cox regression analyses, we found that BAK1, DAPK2, ERO1A, GAPDH, IL1B, ITGA6, NLRC4, NUPR1, SERPINA1, and TOMM40 were significantly associated with OS of LUAD patients. BAK1 belongs to the BCL2 protein family and plays a key role in the mitochondrial apoptotic process. A study on NSCLC showed that miR-150 downregulation can induce cell proliferation inhibition and apoptosis by targeting BAK1 in vitro. Endoplasmic reticulum oxidoreductase 1 alpha (ERO1A) is the major regulator of protein disulfide isomerase (PDI) (Kim et al., 2018). It has been found that co-expression of PDI and ERO1A were independent adverse prognostic factors in NSCLC. NUPR1, also known as p8 and a candidate of metastasis 1 (Sandi et al., 2011), is a transcriptional coregulator that plays regulatory roles in various types of malignant tumors, including pancreatic cancer, multiple myeloma, and bladder cancer (Emma et al., 2016; Ito et al., 2005; Veerla et al., 2008; Zeng et al., 2018). Moreover, NUPR1 expression shows a significant association with OS for NSCLC patients (Mu et al., 2018). The robust autophagy gene prognostic model was established in the training dataset, and validated in the TCGA internal testing dataset. The autophagy signature could classify patients into high-risk and low-risk groups using the median risk score, and patients with high-risk scores had significantly shorter OS than those in the low-risk group. Moreover, another external independent GSE50081 dataset was successfully validated, which indicated a good reproducibility for the signature. Therefore, this autophagy signature may serve as a prognostic biomarker that could potentially be used for clinical application in the future. Our GSEA results suggested that the low-risk group tended to be enriched in the metabolism and immune-related pathways, while the high risk-group was involved in tumorigenesis and tumor progression, and exhibited a strong difference at the pathway level. We also found that LUAD patients in these two groups had distinct immune states. In our study, we noticed that the high-risk group had significantly elevated levels of M0- and M1- macrophages and T cell CD4+memory activated but decreased expressions of CD4 and PD-L1 when compared with the low-risk group. It has been generally accepted that M0, M1 macrophages can produce anti-tumor/pro-inflammatory cytokines, such as reactive oxygen species (ROS) and nitric oxide (NO), to inhibit tumor growth and progression (De Santa et al., 2019). However, M0, M1 macrophage infiltration could also lead to adverse tumor prognosis. For example, a recent study demonstrated that M1 macrophage recruitment correlated strongly with worse OS outcomes in the SHH subgroup of medulloblastoma (Lee et al., 2018). One possible explanation for higher M0, M1 macrophage infiltration in the high-risk group is that autophagy can regulate the tumor immune microenvironment. DAPK2 mediates the formation of autophagic vesicles, which act as a key autophagy regulator (Ber et al., 2015). DAPK2 downregulation can reduce autophagy (Shiloh et al., 2018; Soussi et al., 2015) and could be as a good indicator of autophagic activity. In this study, we observed that DAPK2 expression was downregulated in the high-risk group, suggesting that the autophagic activity was attenuated. It has been reported that cells with attenuated autophagy tend to have higher levels of ROS (Kongara & Karantza, 2012). However, excessive ROS accumulation could activate inflammatory factors such as NF *κ*B, AP-1, and NLRP3 inflammasome, which promote the expression of pro-inflammatory chemokines and cytokines (Harijith, Ebenezer & Natarajan, 2014). Additionally, induced ROS production may trigger the induction of M1-like pro-inflammatory macrophages and regulation of M1 macrophage polarization (Tan et al., 2016). Therefore, autophagy could mediate immune microenvironment reprogramming by altering ROS levels, which affect macrophage polarization. However, due to the complexity of the autophagy and immune response (Jiang et al., 2019), our data provided several implications. Additionally, we developed a nomogram to predict individual prognoses by integrating risk scores and other clinicopathologic features. The nomogram’s performance was established using the whole TCGA-LUAD cohort. The nomogram could provide an accurate OS prediction for LUAD patients.

However, several limitations of this study need to be noted. First, the potential molecular mechanism of the key autophagy genes is not fully understood, and the expressions could be further verified using in vitro or vivo experiments. Second, other LUAD prognostic factors including tumor size, smoking, and lymph node metastasis should be considered. Third, the immune cell fraction in the tumor microenvironment (TME) was quantified using bulk RNA-seq data, and should be validated using more precise methods such as flow cytometry or in situ immunohistochemical imaging (Petitprez et al., 2018).

## Conclusion

In conclusion, our study developed a robust 10-autophagy-related gene signature that could accurately predict OS of LUAD patients. We hope that this prognostic signature could benefit LUAD patients and provide new insights into the underlying mechanisms of this disease.

##  Supplemental Information

10.7717/peerj.11074/supp-1 Figure S1 Expression of classical immune checkpoint markers between low-risk and high-risk group Statistic differences between groups were calculated by Wilcoxon test.

## Additional Information and Declarations

### Data Availability

The following information was supplied regarding data availability: Data are available at the Gene Expression Omnibus (GEO) database: GSE50081. Data is also available at The Cancer Genome Atlas (TCGA): TCGA-LUAD. Our retrieval strategy for TCGA dataset is as follows: in the Cases section, we chose bronchus and lung as Primary Site, TCGA as Program, TCGA-LUAD as Project. In the Files section, we chose transcriptome profiling as Data Category, Gene Expression Quantification as Data Type, RNA-Seq as Experimental Strategy, HTSeq - FPKM as Workflow Type.
