# Supplementary figures and images for: Identification of a novel autophagy signature for predicting survival in patients with lung adenocarcinoma
Source: PeerJ. 2021 Apr 21;9:e11074. doi: 10.7717/peerj.11074 (PMC8067911; doi:10.7717/peerj.11074)

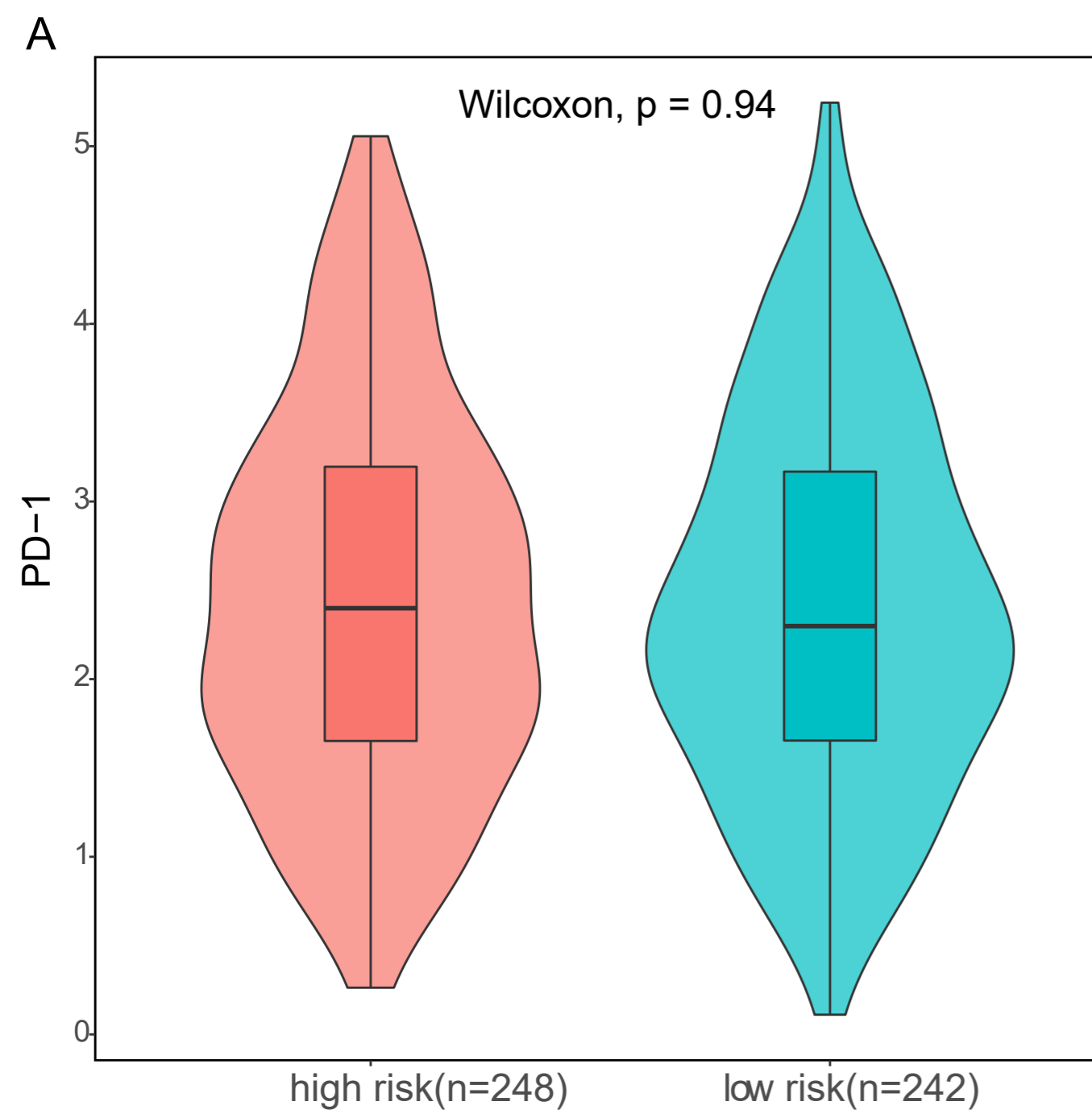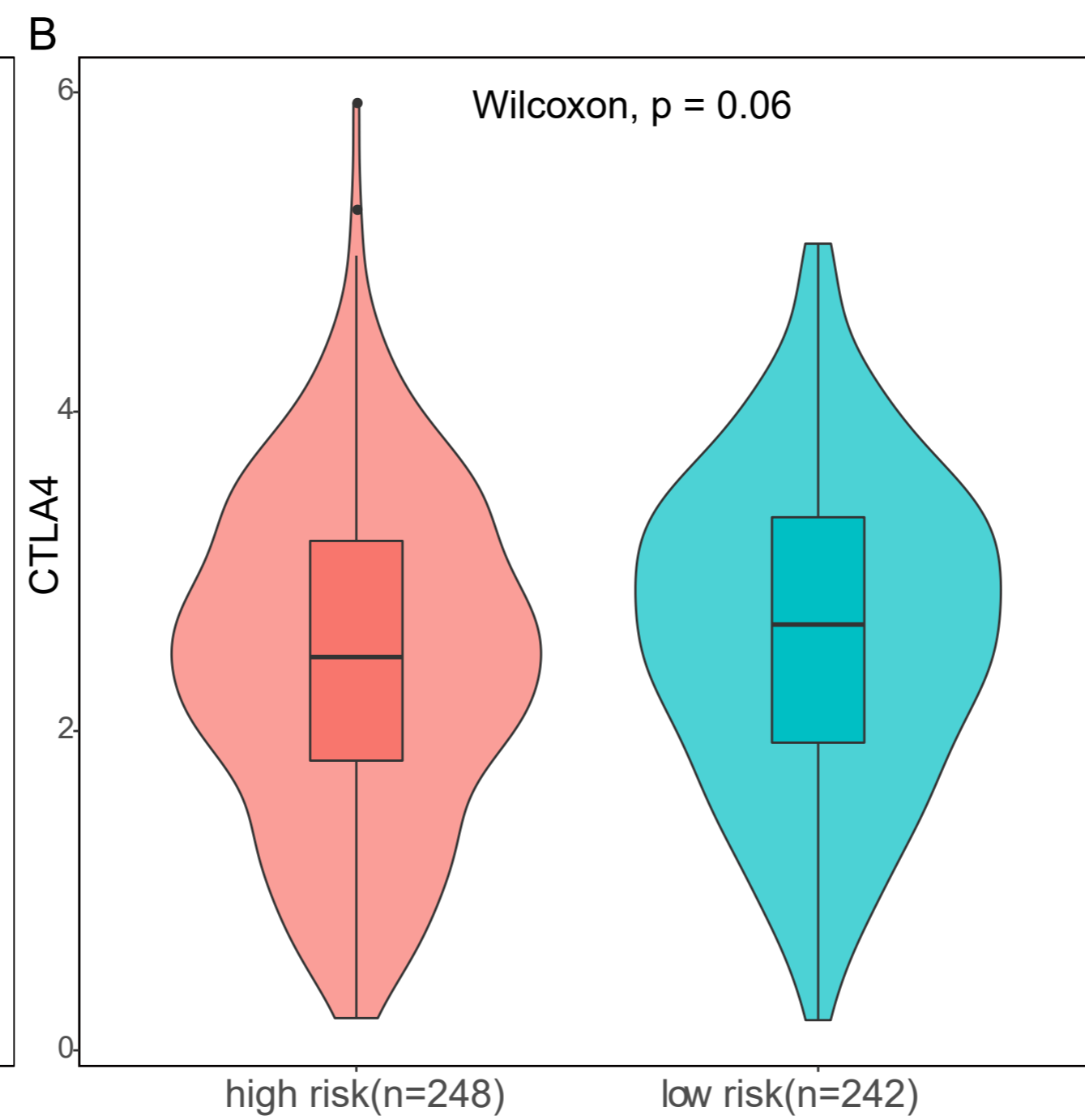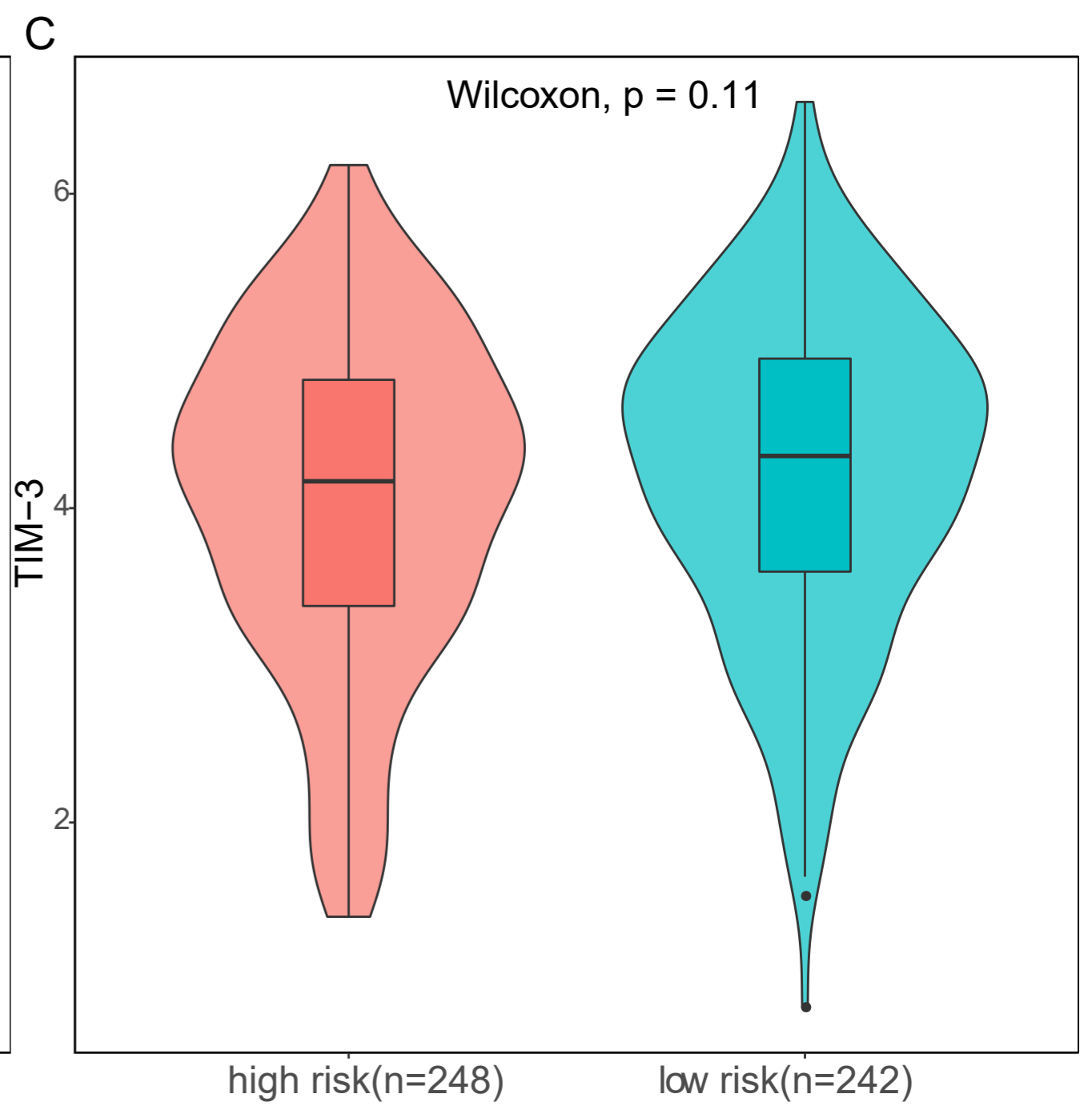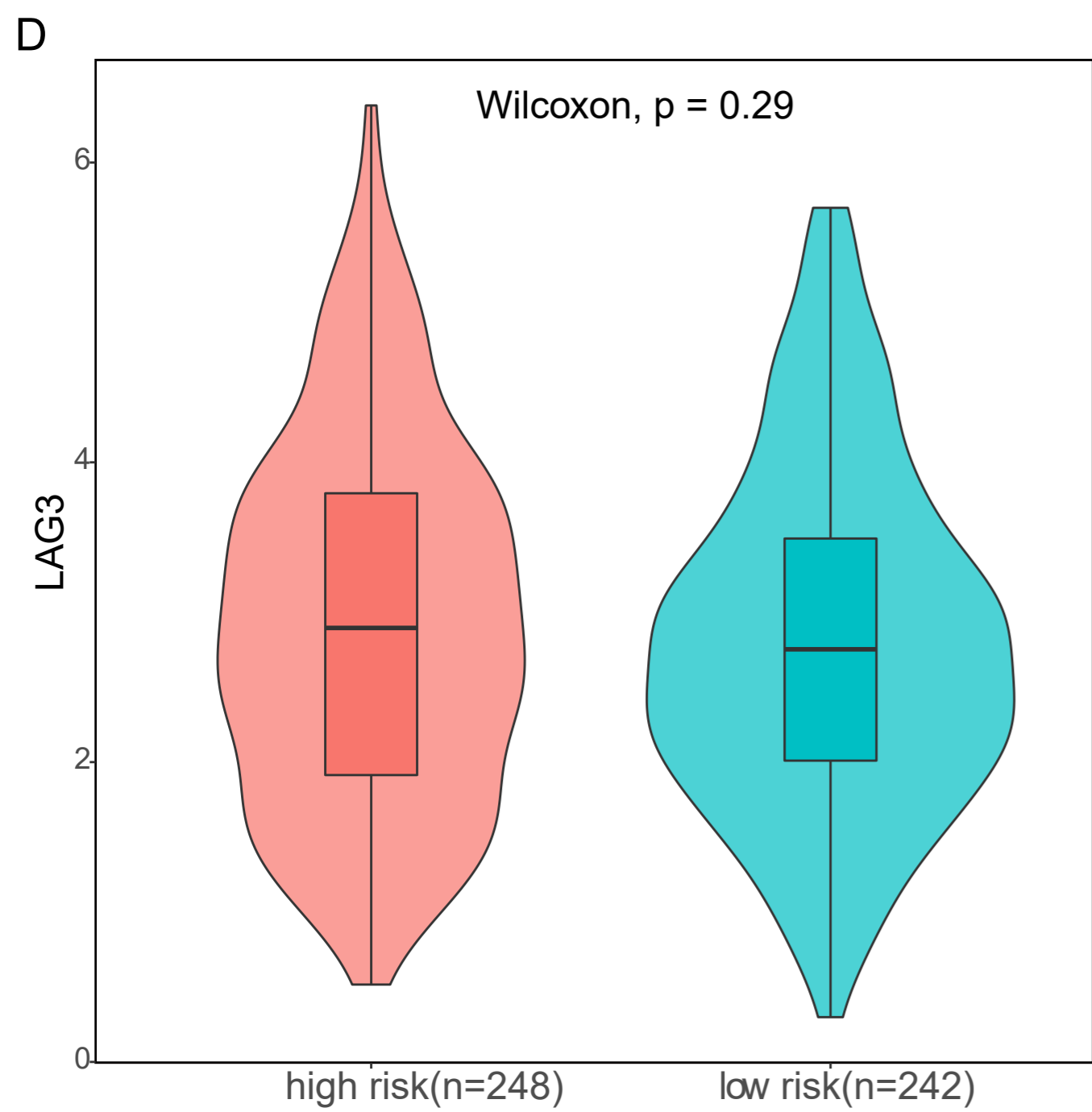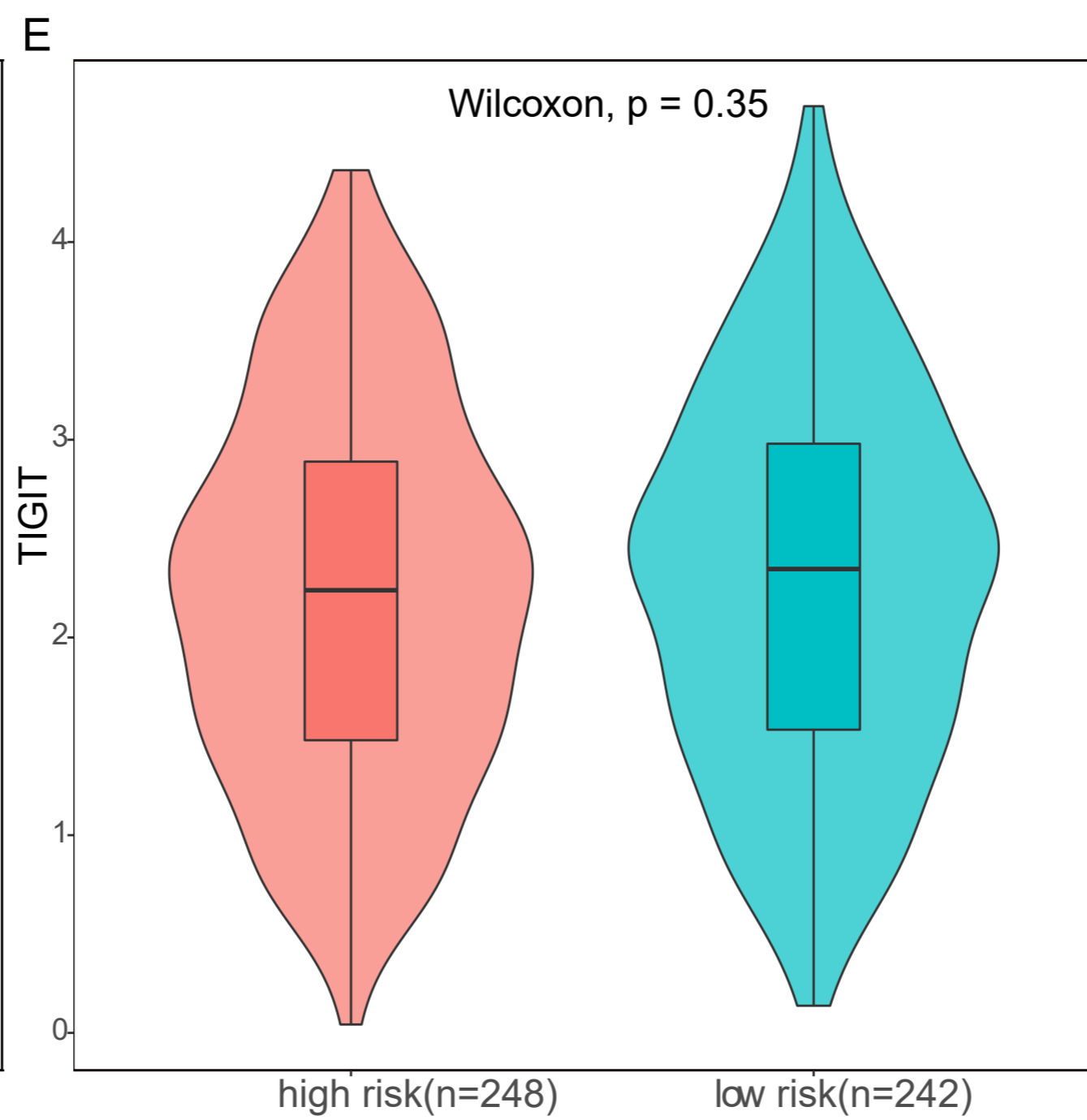

Supplement: Figure S1 — Statistic differences between groups were calculated by Wilcoxon test. [file peerj-09-11074-s001.pdf]
